# Navigating Loss in Animal-Assisted Services: Volunteer Experiences and Implications for Programs Following Therapy Dog Death or Retirement

**DOI:** 10.3390/ani16020202

**Published:** 2026-01-09

**Authors:** Lori R. Kogan, Jennifer Currin-McCulloch, Wendy Packman, Cori Bussolari

**Affiliations:** 1 Department of Clinical Sciences, Colorado State University, Fort Collins, CO 80523, USA; 2 School of Social Work, Colorado State University, Fort Collins, CO 80523, USA; 3 Department of Psychology, Palo Alto University, Palo Alto, CA 94304, USA; wpackman@paloaltou.edu; 4 Department of Counseling Psychology, University of San Francisco, San Francisco, CA 94117, USA

**Keywords:** volunteer role identity, human–animal bond, ambiguous loss, disenfranchised grief, social validation, working dog retirement

## Abstract

Animal-assisted services (AAS) depend on volunteer handler–dog teams, yet the emotional and relational impacts on volunteers when their therapy dog dies or retires remain largely unexplored. This study examines AAS volunteers’ experiences following the death or retirement of their therapy dog partner. An online, anonymous cross-sectional survey was administered between January and June 2025. A total of 247 individual responses were analyzed. Over half of survey participants (56%) had lost a therapy dog to death, and 36.6% had retired a dog. Although most volunteers who resumed AAS with a new dog reported excitement and renewed purpose, many experienced sadness linked to their previous partner. Retirement decisions were primarily driven by dog welfare concerns and were often experienced as an ambiguous loss. Social constraints were common; participants frequently perceived minimization or discomfort from others when attempting to discuss their grief. In conclusion, therapy dog death and retirement represent significant emotional and relational losses for AAS volunteers. Organizational practices, including anticipatory retirement planning, welfare-centered guidelines, recognition rituals, and structured support during successor-dog transitions may help mitigate distress and foster healthy adjustment. Findings are discussed in relation to theory-informed, practical implications for animal-assisted service practitioners and organizations.

## Simple Summary

Animal-assisted services (AAS) rely heavily on volunteer teams of people and their therapy dogs, but very little is known about what volunteers go through emotionally when their dog dies or retires. This study explored those experiences. We conducted an anonymous online survey between January and June 2025. A total of 247 individual responses were analyzed. More than half of respondents (56%) had experienced the death of a therapy dog, and over a third (36.6%) had retired a dog from service. Many volunteers who later worked with a new dog described feeling excited and purposeful again, but also carried sadness connected to their previous partner. Dogs were usually retired to protect their health or well-being, and volunteers often described this transition as challenging. Many also said it was hard to talk openly about their grief because others minimized their feelings or seemed uncomfortable. Overall, the death or retirement of a therapy dog can be a major emotional loss for volunteers. Organizations can help by offering clearer retirement planning, focusing on dog welfare, creating ways to honor retiring or deceased dogs, and providing support during the transition to a new therapy partner. Findings include practical implications for animal-assisted service practitioners and organizations.

## 1. Introduction

Animal-assisted services (AAS) have expanded rapidly across healthcare, educational, and community settings, with numerous studies documenting their benefits in a wide range of settings [1,2,3,4]. AAS can be defined as mediated, guided or facilitator-led practices, programs, and human services that incorporate specially qualified animals into therapeutic, educational, supportive and/or ameliorative processes aimed at enhancing the well-being of humans while ensuring the welfare of the animals involved in these practices [5]. This umbrella term includes animal-assisted treatment, animal-assisted education, and animal-assisted support programs [5]. While much research has been conducted related to client outcomes and increasing animal welfare [6,7,8], less empirical attention has been given to the volunteers who make these services possible.

AAS volunteers participate in a unique triadic relationship involving the handler, their animal partner, and the human clients they serve [9,10], and their work extends beyond typical volunteerism by including a relational, emotional, and welfare-centered framework [11,12]. In a companion paper based on the same dataset (under review), we examined volunteers’ motivations and volunteer role identity using Self-Determination Theory [13] and Role Identity Theory [14]. Self-Determination Theory suggests that people are most motivated and experience greater well-being when their activities are freely chosen and fulfill basic psychological needs for autonomy, competence, and relatedness [13]. Role Identity Theory similarly proposes that roles central to a person’s sense of self carry strong emotional meaning and can, therefore, be quite disruptive when lost [14]. Together, these frameworks help explain why AAS volunteering—when it is intrinsically motivated and closely tied to identity—can make changes to the human–animal partnership emotionally and psychologically consequential. That study demonstrated that AAS volunteers are primarily driven by autonomous, value-based motivation and an internalized sense of identity in their role, and that engagement in AAS is strongly linked to enhanced well-being and purpose. Importantly, prior work suggests that volunteer role identity in AAS is often deeply anchored in the human–animal bond, suggesting that volunteers’ motivations, sense of purpose, and self-concept are closely intertwined with their relationship to their dog [15].

These findings underscore that AAS volunteers’ relationships with their dogs are central to their motivation, identity, and sense of meaning. Given this context, the loss of a therapy dog through death or retirement represents not only the end of a relationship but also a potential disruption to volunteers’ sense of purpose, identity, and routine. Yet, despite the significant impact of this relationship, little research has explored what happens when these partnerships end.

Losing a companion animal often elicits grief responses comparable to the loss of a human loved one [16,17,18,19], yet grief after pet loss is often disenfranchised—socially minimized and unrecognized [17,18,20]. These challenges may be amplified for AAS volunteers, whose relationships with their dogs include shared caregiving roles, emotional labor, and a sense of joint purpose. When the foundation of volunteer motivation and role identity are grounded in the relationship with their dog, the loss of that dog represents not only relational bereavement but also a disruption to a meaningful volunteer identity. Therapy dog retirement includes its own unique aspects. Because the dog remains alive but is no longer able to participate in AAS, the loss may be ambiguous and difficult to articulate [21,22]. Ambiguous loss often creates “frozen grief,” in which the absence of closure, ritual, or clear social acknowledgment complicates emotional processing [23,24]. Prior work suggests that service-animal retirement, in addition, can create identity disruption, uncertainty, and emotional strain [18,25].

Adjustment to therapy dog loss, whether through death or retirement, may be negatively impacted by social constraints (i.e., the reactions by others that discourage emotional expression) [26]. Social constraints are associated with poorer psychological outcomes across stressful experiences [27,28,29,30,31], and individuals grieving companion-animal loss frequently report feeling dismissed or misunderstood [32,33,34,35]. Research across caregiving fields, volunteer identity theory, and companion-animal grief suggests that for individuals engaged in AAS, the absence of social validation during their loss could amplify feelings of disenfranchised grief and negatively impact psychological adjustment [20,32,36,37,38].

Despite these potentially profound effects, the retirement or loss of therapy dogs has received minimal attention. Existing research on working dog loss has largely focused on military and service dog contexts [16,18,39,40,41,42,43], with very few studies exploring the loss or retirement of therapy dogs [44]. Other working-dog roles, such as police K-9 partnerships, may be particularly vulnerable to constrained grief and limited social validation due to occupational cultures emphasizing stoicism and instrumental role framing; however, empirical research in this area remains sparse [45,46]. The present study addresses this gap by examining AAS volunteers’ experiences of losing a therapy dog partner, through either death or retirement, and the emotional, relational, and social processes that accompany this transition. Our goal is to provide actionable insights for AAS programs to better support therapy dog teams before, during, and after the loss or retirement of a therapy dog partner.

Although the present study focuses on psychological, relational, and social experiences of loss, it is important to situate these experiences within a broader biopsychosocial context. Loss experiences, including bereavement, have been associated with physiological stress responses that may affect health and well-being. Prolonged or ambiguous grief can be accompanied by activation of stress-related biological systems, including the hypothalamic–pituitary–adrenal (HPA) axis and alterations in glucocorticoid activity [47,48], which have been linked to changes in emotional and cognitive processes [49,50,51]. While physiological outcomes were not assessed in the present study, recognition of these processes underscores the importance of social and organizational support that may buffer stress and support adjustment following therapy dog death or retirement.

The present study was grounded in Self-Determination Theory and Role Identity Theory, which together led to several theory-based expectations about volunteers’ responses to therapy dog loss and retirement. Because AAS volunteering is often intrinsically motivated and fulfills core needs for relatedness and purpose, we expected the loss or retirement of a therapy dog to be emotionally significant. Further, when a volunteer role is closely aligned with one’s sense of self and anchored by the human–animal bond, disruption of that role was expected to contribute to experiences of ambiguous or prolonged grief, particularly in the absence of social recognition or validation. Finally, consistent with role identity theory, we anticipated that volunteers’ adjustment following their loss would be shaped not only by the death or retirement itself, but also by the amount of social support and opportunities for continued role engagement.

## 2. Materials and Methods

An online, anonymous, cross-sectional survey was developed using Qualtrics (https://www.qualtrics.com/, Qualtrics, Inc., Provo, UT, USA). This study was approved by the Colorado State University Institutional Review Board (IRB #6446). Participants were recruited between January and June 2025 through the social media platforms Facebook and Instagram, as well as through newsletters distributed by AAS organizations. A total of 247 participants completed the survey and were included in the analyses. The survey introduction described the purpose of the research, obtained informed consent, and provided instructions for respondents with more than one dog. The broader survey was designed to examine two interconnected domains of AAS volunteer experience: (1) volunteers’ motivations and identities related to AAS participation, and (2) the grief, adjustment, and social responses experienced when a therapy dog retires or dies. The present study focuses on the experiences of loss through dog retirement or death.

The survey began with screening questions to determine whether participants currently provide, previously provided, or had never provided AAS, followed by items related to AAS involvement. These included the number of dogs trained and type of training, duration of volunteer service, dog certification status, and settings in which visits were conducted. Additional items assessing perceived personal and relational benefits of AAS participation and motivational constructs (e.g., autonomous versus controlled motivation, volunteer role identity) were also collected but are reported in the previous companion paper.

For the purpose of the present study, analyses centered on respondents’ experiences following the loss of a therapy dog through either death or retirement. Participants first indicated whether they had ever lost a dog partner to death and, if so, how long ago the most recent death occurred. Those who had lost more than one dog were instructed to respond according to their most recent loss. Participants then reported whether they had resumed AAS work with a new dog and completed a series of 5-point Likert-type items (1 = strongly disagree, 5 = strongly agree) assessing their emotional experiences during this transition, including tendencies to compare the new dog to the previous one, feelings of guilt or frustration during retraining, sadness when engaging in therapy work, and feelings of excitement or renewed purpose.

Another set of questions assessed experiences related to therapy dog retirement. Respondents indicated whether they had ever retired a therapy dog, when the most recent retirement occurred, and the reasons for this decision, including behavioral changes, medical issues, aging, lifestyle changes, or lack of enjoyment. Participants also evaluated whether they believed the retirement occurred too early, at the right time, or later than ideal.

To understand the interpersonal context surrounding these losses, respondents completed the Social Constraints Measure (SCS), a 15-item instrument evaluating the extent to which friends and family members responded to the participant’s grief in ways that discouraged open emotional expression [52]. The items asked about experiences such as others minimizing or trivializing the loss, changing the subject, appearing uncomfortable, or giving the impression that the participant should not discuss the death or retirement. Items were rated on a four-point scale ranging from “never” to “often,” and total scores reflected the degree of perceived constraint in discussing the loss. Internal consistency in the current sample was excellent for both death-related (α = 0.953) and retirement-related (α = 0.957) experiences. Because respondents could report on death, retirement, or both—and these experiences were not uniformly paired, SCS data were analyzed descriptively rather than through inferential comparisons.

Participants also responded to open-ended questions inviting them to describe what would have been most helpful during their adjustment process for both death and retirement, and to share any additional information that would further elucidate their loss experiences.

### 2.1. Statistical Analysis

Descriptive statistics and frequency distributions (reported as percentages) were computed with IBM SPSS, version 31, Armonk, NY, USA. Because not all questions were answered by all respondents, the total number of respondents for each question varied. Reported percentages for each question are based on total responses for that question.

### 2.2. Qualitative Analysis

Thematic analysis methods [53] were utilized to determine themes among the open-ended survey data. Thematic analysis is a systematic qualitative method used to identify, analyze, and interpret recurring patterns of meaning (“themes”) within textual data. Rather than quantifying responses, this approach focuses on understanding how participants describe and make sense of their experiences, making it well-suited for exploring complex emotional, relational, and contextual processes [53]. In the present study, thematic analysis was used to examine open-ended responses describing volunteers’ experiences following therapy dog death or retirement and their perceptions of what would have been most helpful during these transitions. An inductive approach was employed, meaning that themes were derived from the data themselves rather than imposed a priori. This method allows participants’ perspectives to guide interpretation while maintaining analytic rigor through systematic coding, comparison across responses, and refinement of themes.

This six-step inductive analytic process sought to determine salient themes and quotes to represent handlers’ adjustment to loss and advice about ways to best support them during loss transitions. The steps include (1) gathering an overall understanding of the data; (2) determining initial codes in the data; (3) looking for patterns in the data; (4) portraying themes from these patterns of data; (5) determining definitions and names for themes; and (6) presenting findings.

## 3. Results

A total of 247 individuals participated in this study. Participants predominantly identified as women (89.8%), with smaller proportions identifying as men (8.0%) or other (2.2%). Most participants were partnered or married (69.9%). The sample was largely White/Caucasian (88.8%) and not Hispanic/Latinx (88.6%). A total of 69.4% of respondents were 50 years or older. Participants were highly educated, with 70.0% reporting a graduate or professional degree. Household income varied, with the largest number of participants reporting incomes between $70,000 and $99,999 (23.8%) or $150,000 or more (22.0%) (Table 1).

**Figure 1:**
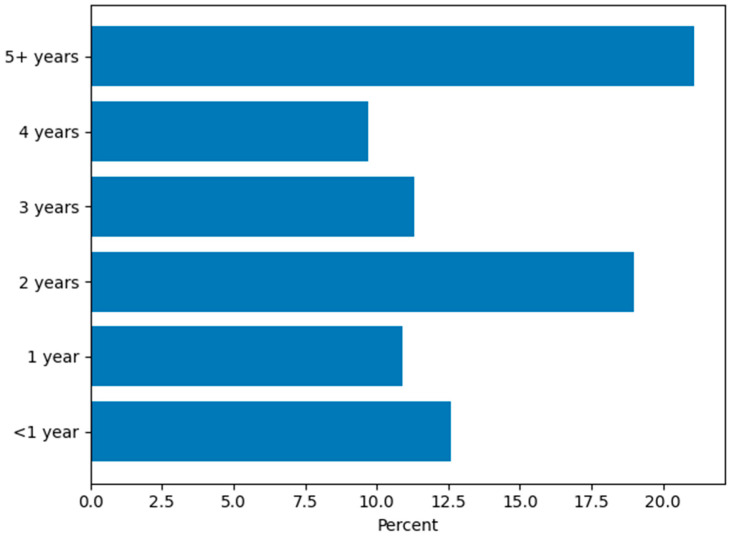
Length of Time Conducting AAS.

**Figure 2:**
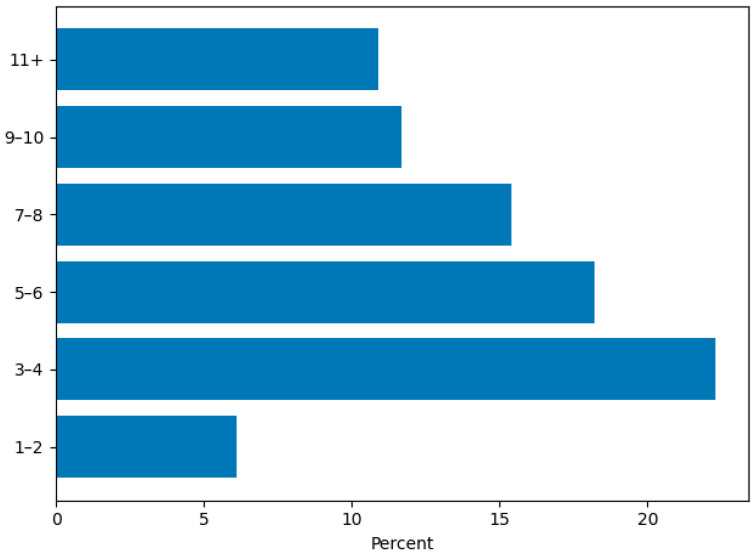
Age of Therapy Dogs.

**Figure 3:**
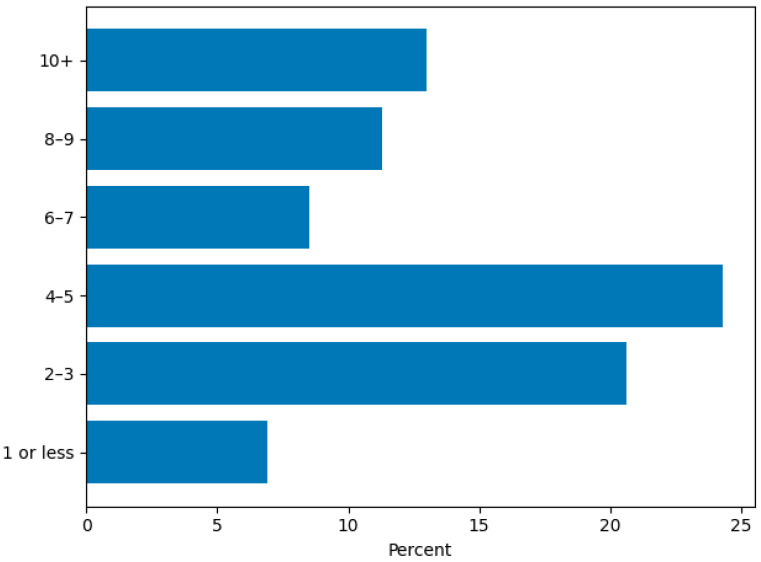
Days per Month Providing AAS.

**Figure 4:**
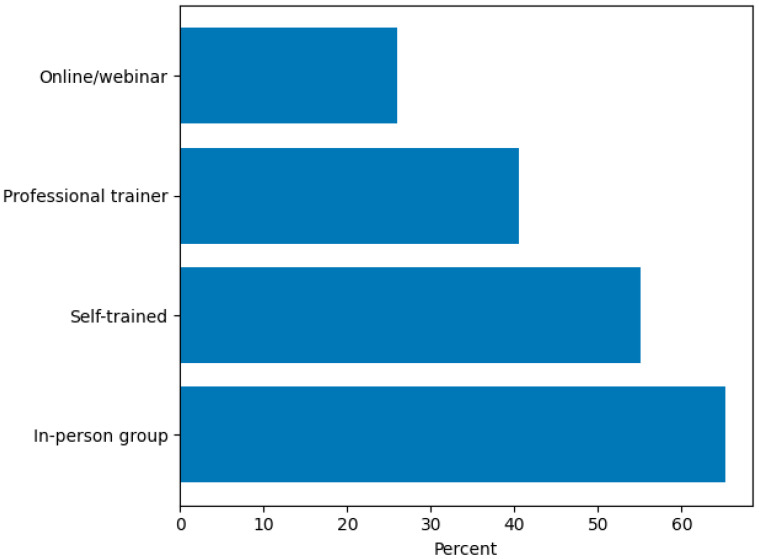
AAS Service Settings (Multiple Responses Possible).

The majority of participants (202/247; 81.8%) indicated that they currently provide AAS with their dog, with approximately one in five (52/247; 21.1%) indicating they had been conducting AAS for five or more years (Figure 1). Most therapy dogs were between 3 and 8 years of age (138/247; 55.9%) (Figure 2), and 103/247 (41.7%) of respondents indicated they acquired their dog specifically for AAS (Supplementary Table S1). Two-thirds 163/247 (66.0%) were providing AAS with one dog, and 34/247 (13.8%) with two dogs. Regarding time commitment, most volunteers provided AAS 2–5 days per month (111/247; 44.9%), while 32/247 (13.0%) engaged in AAS 10 or more days per month, and 17/247 (6.9%) reported providing AAS one day per month or less (Figure 3). Participants recounted conducting AAS across a broad range of settings. The most common environments included K–12 schools (116/247; 47.0%), nursing homes (95/247; 38.5%), hospitals (109/247; 44.1%), colleges or universities (91/247; 36.8%), and libraries (69/247; 27.9%). In addition, 112/247 (45.3%) reported “other” settings such as mental health clinics, hospice programs, airports, community events, and private practice (Figure 4). The majority (191/247; 77.3%) reported that their dog was certified or registered as a therapy dog (Supplementary Table S1).

**Figure 5:**
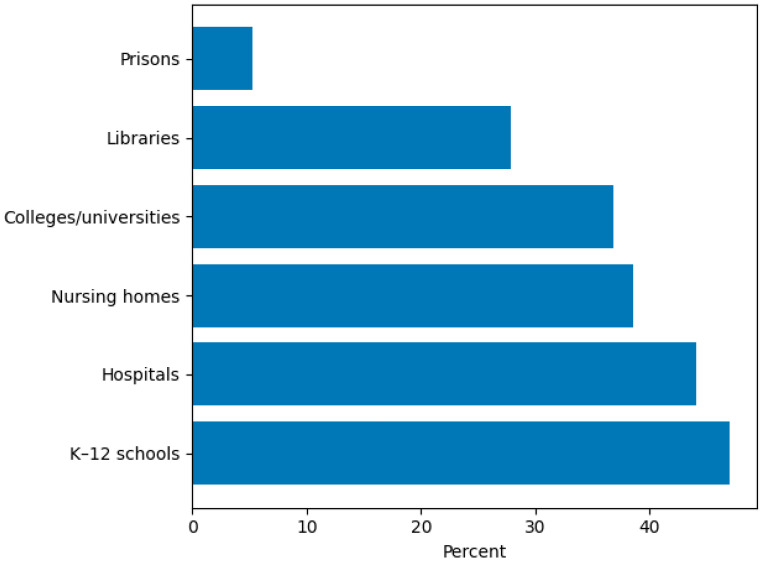
Training Pathways for Therapy Dogs (Multiple Responses Possible).

Nearly half of the sample (112/247; 45.3%) had trained only one AAS dog at the time of the survey (Supplementary Table S1). When asked about types of training, 161/247 (65.2%) indicated they had attended in-person group classes, 136/247 (55.1%) had self-trained their dogs, 100/247 (40.5%) reported working with an individual professional trainer, and 64/247 (25.9%) participated in online or webinar-based training (Figure 5).

### 3.1. Therapy Dog Death

A total of 131/234 respondents (56.0%) indicated they had lost a therapy dog due to death. Among those, most reported that the loss had occurred three years ago or longer (71/128; 55.5%) and that they had resumed AAS work with a new dog (99/126; 78.6%). Those who resumed AAS work were asked to report their feelings about working with a new dog. Overall, volunteers described predominantly positive experiences. For example, 79/95 (83.2%) reported disagreeing that they had less patience with their new dog, 82/95 (86.3%) disagreed that they felt guilty about training their new dog, and 76/95 (80.0%) disagreed that they felt frustrated about starting over. However, sadness linked to the previous dog remained present for some, with 23/95 (24.2%) indicating that they felt sad when thinking about their prior therapy dog during AAS sessions. In contrast, experiences of excitement and renewed purpose were strong. Almost all respondents, 89/95 (93.7%), reported feeling excited to be doing therapy again, and felt a sense of renewed purpose (66/94, 70.2%) (Table 2).

### 3.2. Social Constraints Measure (SCS)—Death

The mean sum of the 15 SCS items was 24.87 (SD = 9.14, range 15–55). This can be compared to a previous study of service dog handlers who lost a dog through death (M = 22.72, SD = 10.3) [18].

When asked what would have been the most helpful after the death of their therapy dog (*n* = 101), the most common responses were obtaining a new dog (*n* = 26, 26%), support from friends and family (*n* = 20, 20%), memorials, validation and recognition (*n* = 19, 19%), and peer/handler support (*n* = 10, 10%) (Table 3).

Thematic analysis determined four themes among the handlers’ responses (*n* = 101) to what has been most helpful after the death of their therapy dog (Table 3). These include the benefits of obtaining a new dog and having canine companionship; the support of family and friends; memorialization processes; and receiving support from peers who have experienced the loss a therapy dog.

### 3.3. Retirement

A total of 83/227 (36.6%) participants indicated they had retired a therapy dog. Most of these respondents reported that the retirement had occurred at least 3 years ago (55/83, 66.2%). The most common reasons cited to retire their dog were related to the dog’s condition, including old age (46/83, 55.4%) and medical issues (36/83, 43.4%). A smaller number of respondents indicated that their dog no longer seemed to enjoy therapy work (21/83, 25.3%) or experienced behavioral or stress-related difficulties (14/83, 16.9%). Handler-related factors—including the volunteer’s own health (5/83, 6.0%), lifestyle changes (4/83, 4.8%), or lack of opportunity (1/83, 1.2%)—were less frequently endorsed (Table 4). The majority of respondents (76/83, 91.6%) felt confident they made the retirement decision at the right time, compared to 3/83 (3.6%) who felt they retired their dog too early and 4/83 (4.8%) who felt they retired their dog too late.

### 3.4. Social Constraints Measure (SCS)—Retirement 

The mean sum of the 15 SCS items was 23.26 (SD = 9.60, range 15–55). This can be compared to a previous study of service dog handlers who lost a dog through retirement (M = 20.94 (SD = 9.45) [18].

When asked what would have been the most helpful after the retirement of their therapy dog (n = 83), the most common responses were having another dog or training a new dog (n = 33), support from friends and family (n = 14), and other handlers/AAS community (n = 13) (Table 5).

Similar to findings from respondents’ experiences with therapy dog death, retirement themes (Table 5) included finding another dog, memorialization processes, family and friends, and AAS peers. However, three novel themes were identified in respondents’ reports of what would have been most helpful after the retirement of their therapy dog (*n* = 83), including confidence in their decisions, structured retirement planning, and staying involved in AAS.

When asked what else would be helpful for others to know about the loss of their therapy dog partner, responses emphasized the importance of acknowledging and validating their loss, compassionate listening, and the importance of recognizing their dog’s contributions. Several described supportive gestures such as retirement parties, sympathy from staff and children, or inclusion in newsletters. They felt these forms of recognition helped them feel others understood the significance of their loss.

Participants also commonly referenced the deep emotional impact of losing a therapy dog, often describing the loss as profound and one of the most difficult they had experienced (“I have never experienced a loss… that has affected me as much as losing my therapy dog”). Many highlighted the unique relationship, noting how much they missed the routines and shared experiences of AAS work. Another dominant theme related to ethical decision-making and the prioritization of their dog’s welfare. Handlers repeatedly stressed their responsibility in recognizing subtle signs of stress, pain, or decreased enjoyment. Several described making retirement decisions proactively, sometimes despite external pressure to continue. These reflections underscored the centrality of animal welfare in participants’ retirement decisions.

Several respondents described how other dogs in their household provided comfort or helped maintain a sense of continuity after their loss. At the same time, some mentioned struggling with comparing their new dogs to their previous partners, acknowledging the challenges of transitioning to a new working relationship (“It is very hard not to compare one dog to another;” “It is difficult to follow up an amazing partner”).

Participants also discussed the loss of not only the dog but also of the shared role and purpose associated with AAS work. Many missed the therapy visits and interactions (“Time spent with my therapy dogs and visiting patients was often the highlight of each week”), while others detailed ways they had been able to stay connected to AAS through alternative forms of involvement.

## 4. Discussion

The present study advances the understanding of AAS by examining the experiences of volunteers following the death or retirement of their therapy dog partner. While our companion paper (under review) demonstrates that AAS volunteers are primarily driven by autonomous, value-based motivation and strong role identity, the current study reveals how those same motivations and identities shape volunteers’ experiences when a partnership ends. Together, these two lines of research highlight that AAS volunteerism involves meaningful identity anchored by the human–animal bond. Viewed through this lens of volunteer motivation and role identity, the grief responses observed in the present study are unsurprising: when service is intrinsically motivated and integrated into one’s sense of self, the loss of a dog partner who enables that service can generate both relational grief and role-based loss, helping to explain experiences of prolonged distress and grief.

Consistent with Self-Determination Theory [13], volunteers in our companion study described therapy dog work as an internalized, personally meaningful activity that positively impacts their sense of purpose, happiness, and overall well-being. This provides important context for understanding the profound impact of losing a therapy dog partner. When a volunteer’s work is rooted in intrinsic enjoyment, personal values, and a strong relational bond with their dog, the loss of that dog represents not just the end of an activity but a disruption of identity, routine, and meaning.

Participants’ open-ended responses reflected this sentiment. Many described the loss as unlike any other they had experienced, noting it was “one of the hardest things” they had gone through, or that “I am over 60, and have never experienced a loss of a person or pet that has affected me as much as losing my therapy dog.” This mirrors prior research on the intensity of companion-animal grief [17,20,54] as well as the uniqueness of grief following the loss of a working partner [18,25].

Results from this study also align with the findings of our companion study in which volunteers endorsed strong animal-centered motives and described therapy work as rewarding for both them and their dogs. When retirement or death occurs, this shared purpose is interrupted. Several participants expressed grief not only over losing their dog but also losing the meaningful routines of service: “I miss the time spent together in all of those places,” and “Time spent with my therapy dogs and visiting patients was often the highlight of each week.” Several participants explicitly noted the dual nature of their grief, stating, “You may mourn the loss of the partnership and services you provided. You miss the dynamic you once had with your pet.” These comments capture the entwined emotional, relational, and identity-based aspects of therapy dog loss. Such reflections support theoretical models that suggest when people derive life purpose and identity from a role, the disruption of that role can often create distress and negative psychological impacts [38,55].

Retirement of therapy dogs poses unique emotional challenges and can perhaps be best understood through the lens of ambiguous loss [21], in which a loved one is physically present but psychologically absent from the role they once occupied, making closure difficult. This loss can be made even more difficult when volunteers feel pulled between external expectations and their commitment to their dog’s welfare. As one participant explained, “People need to pay attention to their dogs… often something has changed for the dog, and they no longer enjoy the work.” Another described feeling torn when their decision to retire was questioned: “I had pressure from families because they didn’t understand why I chose to retire him… they were well-meaning but felt let down.” For some volunteers, this pressure led to feelings of doubt, as two participants explained, “She still did want to work, but she had weak back legs, and I was worried she would fall,” and “I haven’t really reconciled retiring her yet. I keep hoping I will find one more situation where we could visit together.”

The Social Constraints Measure results in our study further reinforce the complex interpersonal dynamics surrounding therapy dog loss. Many participants reported experiencing minimization, discomfort, or avoidance from others when discussing their loss; patterns documented in previous pet loss research [32]. Such social constraints can negatively impact an owner’s ability to emotionally process their loss and contribute to feelings of isolation.

Although some participants struggled with their loss, others described factors that facilitated their coping. Peer, family, and friend support emerged as particularly meaningful. Organizational rituals, such as newsletters honoring retired or deceased dogs, were also valued. These practices echo research on meaning-making and identity affirmation as key components of healthy adjustment following role transitions and bereavement [56,57,58].

The transition to working with a new therapy dog, although primarily viewed as a positive choice, was challenging for some participants. Some described the difficulty in not comparing their new dog to their previous dog: “It is very hard not to compare one dog to another, especially when one is such a great therapy dog,” while others noted the difficulty of following “an amazing partner with one who is not as interested in the work.” Yet many found comfort from working with a new therapy dog: “It takes a long time for the pain to subside, but the other dog(s) are a comfort.” Several participants in the current study described the comfort, continuity, and sense of meaning that came from having a new dog in training, consistent with findings in working dog fields where succession planning supports smoother transitions [40,41,59].

### 4.1. Theory-Informed Implications and Directions for Future Research

Together, findings from both this study and the companion study offer practical implications for therapy dog organizations. Because volunteers are driven by autonomous, value-based motivations and view their AAS role as core to their identity, organizations should anticipate that the death and/or retirement of a therapy dog will be an emotionally significant event. The following implications are offered as theory-informed and practice-relevant considerations.

Programs should consider implementing anticipatory grief guidance as part of initial volunteer training. Programs can help address this by:

- Creating standardized retirement guidelines that outline behavioral, physical, and emotional indicators suggesting that a dog is no longer enjoying the work. These guidelines should be informed by animal welfare research demonstrating that subtle stress cues are often missed by humans [60,61,62].
- Offering retirement-readiness checklists at annual evaluations that include veterinary assessments, handler observations, and welfare-based criteria.
- Initiating anticipatory retirement conversations early, especially as dogs age or shift into more demanding settings. This mirrors recommendations in service-dog research, where structured planning has been shown to reduce emotional distress and improve decision-making [40,41].
- Providing educational resources (e.g., webinars, infographics, and case examples) to help handlers understand typical aging trajectories, behavioral changes, and the importance of assessing welfare and quality of life.
- Encouraging volunteers to train or identify potential successor dogs before the retirement of their current therapy dog.

#### 4.1.1. Recognize and Honor Retirement

The literature on grief rituals suggests that marking transitions can foster meaning-making and emotional adjustment [63,64,65]. To this end, it is suggested that programs:

- Offer retirement ceremonies or certificates to acknowledge the dog’s contributions.
- Provide sample scripts or templates that handlers can use to inform clients and facilities about the transition.
- Facilitate opportunities for clients or facility staff to express gratitude (e.g., letters, drawings, group acknowledgments, etc.).

These practices can help validate the significance of the loss, reduce social constraints, and promote a sense of closure.

#### 4.1.2. Supporting Handlers After Loss—Death or Retirement

AAS programs are uniquely positioned to reduce handlers’ feelings of isolation and foster supportive environments, which can reduce feelings of distress following their loss [66,67]. To this end, we suggest organizations offer the following programs:

- Establish peer-support networks, such as small discussion groups, mentorship relationships, or online spaces where handlers can share stories and obtain validation from others who understand the unique nature of AAS-related grief.
- Partner with veterinary social workers or other mental health professionals to offer grief-informed workshops or resources.
- Create “legacy spaces” (e.g., digital memorial sites, newsletters, etc.) that honor retired or deceased therapy dogs and affirm the dogs’ contributions.
- Check in with volunteers after retirement or death, immediately afterward, as well as periodically, acknowledging that grief can be delayed or long-lasting.
- Encourage handlers to remain involved in other program roles (e.g., mentor new volunteers, assist with evaluations, participate in event planning, etc.), which can help them maintain identity and purpose during their transition period.

#### 4.1.3. Encouraging Dog-Centered Decisions

Some participants described pressure from clients or facility staff to continue working despite behavioral or physical signs indicating that their dog was no longer enjoying sessions. Prior research suggests that handlers can overestimate client expectations and underestimate dog-related welfare concerns [41]. To address these issues, it is suggested that programs:

- Adopt policies explicitly stating that dog welfare supersedes service demands, reinforcing that it is both acceptable and expected to prioritize the dog’s well-being.
- Train facilities and partner organizations to understand that participation is conditional, based on the dog’s enjoyment and welfare, and help them proactively plan for the dog’s retirement.

#### 4.1.4. Preparing Handlers for Successor Dogs

Transitioning to a new therapy dog can be a positive experience, but it can be complicated by comparisons to a previous dog, guilt, or unrealistic expectations. Successor transitions in working-dog contexts benefit from social support, expectation-setting, and emotional acknowledgment [40,41,59]. To this end, programs can:

- Offer educational materials on successor-dog adjustment that normalize mixed emotions (e.g., excitement, sadness, guilt, etc.).
- Provide mentorship for handlers navigating the transition, particularly those working with their first successor dog.
- Encourage gradual integration, allowing handlers to build confidence with a new dog without feeling rushed to reestablish previous routines.

Future research is needed to empirically test the effectiveness of these theory-informed strategies, including whether they reduce social constraints, support volunteer well-being, or promote welfare-centered decision-making over time.

### 4.2. Limitations

Several limitations should be considered when interpreting the findings of this study. First, the use of an online, self-selected sample recruited through social media and AAS organizational newsletters may have introduced selection and expectancy biases, as volunteers who are highly engaged in AAS or who view their experiences positively or meaningfully may have been more likely to participate. This recruitment approach may also have attracted individuals who were particularly motivated to reflect on or share their loss experiences. The sample was also predominantly composed of long-term, highly committed volunteers, which may limit generalizability to individuals with more limited AAS involvement or more diverse demographic backgrounds.

Second, this study relied on retrospective self-reports, which introduces the potential for reporting and recall biases. Participants’ descriptions of their emotional experiences following therapy dog death or retirement may have been shaped by current perspectives and emotional processing over time. Finally, because the data capture a single time point, this study cannot assess how distress, adjustment, or perceptions of support may change over time. Future research using longitudinal designs, more diverse samples, and mixed-method or observational approaches could further elucidate how therapy dog loss unfolds over time and how programs can best support volunteers during these transitions.

## 5. Conclusions

Together, the combined findings from our previous study and the present analysis of loss experiences underscore that therapy dog volunteerism is a meaningful, identity-shaping activity grounded in intrinsic motivation and the human–animal bond. When a therapy dog retires or dies, volunteers experience a myriad of feelings, including grief, role loss, and disrupted purpose. AAS programs can support volunteers during these challenging times in ways that protect animal welfare and sustain long-term engagement through anticipatory planning, welfare-centered guidance, formal and informal recognition, and ongoing emotional support.

## Figures and Tables

**Table 1:** Participant Gender Identity, Race, Ethnicity, Education Level, Household Income, Relationship Status, and Age.

Variable	Category	*N*	Percent
Gender Identity (*n* = 225)	Woman	202	89.8
	Man	18	8.0
	Nonbinary	1	0.4
	Two Spirit	1	0.4
	Prefer not to say	3	1.3
Race (*n* = 224)	White/Caucasian	199	88.8
	Asian	7	3.1
	Biracial/Multiracial	5	2.2
	Middle Eastern	1	0.4
	Native American/Indigenous	1	0.4
	Native Hawaiian/Pacific Islander	1	0.4
	Prefer not to say	10	4.5
Ethnicity (*n* = 219)	Hispanic/Latinx	8	3.7
	Not Hispanic/Latinx	194	88.6
	Prefer not to say	17	7.8
Education Level (*n* = 226)	≤High school/GED	3	1.3
	Vocational/trade/2-year degree	16	7.1
	College (4-year degree)	47	20.8
	Graduate/professional degree	154	68.1
	Prefer not to say	6	2.7
Annual Household Income (*n* = 224)	<$10,000	5	2.2
	$10,000–$29,999	7	3.1
	$30,000–$49,999	12	5.4
	$50,000–$69,999	35	15.6
	$70,000–$99,999	40	17.9
	$100,000–$149,999	32	14.3
	≥$150,000	37	16.5
	Prefer not to say	56	25.0
Marital/Relationship Status (*n* = 226)	Single/Divorced/Widowed	66	29.2
	Partnered/Married	153	67.7
	Prefer not to say	7	3.1
Age (*n* = 225)	Under 30 years	10	4.4
	30–39 years	19	8.4
	40–49 years	39	17.3
	50–59 years	45	20.0
	60–69 years	64	28.4
	≥70 years	45	20.0
	Prefer not to say	3	1.3

**Table 2:** Emotional Responses to Working with a New Therapy Dog after the Death of a Previous Dog.

Item	Strongly Disagree	Somewhat Disagree	Neither Agree nor Disagree	Somewhat Agree	Strongly Agree	N
I tend to have less patience with my new dog.	65/95 (68.4%)	14/95 (14.7%)	10/95 (10.5%)	4/95 (4.2%)	2/95 (2.1%)	95
I often feel guilty about training my new dog.	77/95 (81.1%)	5/95 (5.3%)	8/95 (8.4%)	4/95 (4.2%)	1/95 (1.1%)	95
I feel frustrated about having to start over.	66/95 (69.5%)	10/95 (10.5%)	13/95 (13.7%)	4/95 (4.2%)	2/95 (2.1%)	95
I feel sad thinking about my old dog when doing therapy with my new dog.	46/95 (48.4%)	13/95 (13.7%)	13/95 (13.7%)	19/95 (20.0%)	4/95 (4.2%)	95
I feel excited to be doing therapy again.	1/95 (1.1%)	0/95 (—)	5/95 (5.3%)	23/95 (24.2%)	66/95 (69.5%)	95
I feel a renewed sense of purpose.	2/94 (2.1%)	0/94 (—)	26/94 (27.7%)	22/94 (23.4%)	44/94 (46.8%)	94

**Table 3:** Thematic Analysis of Handlers’ Perceived Supports Following Therapy Dog Death.

Theme	Representative Quotes
Obtaining a New Dog/Canine Companionship	“For all of my adult life, I have lived with multiple canine companions… the comfort providing one another helps the healing process.” “I am fortunate that my late partner helped raise my new one… it gave me comfort when I had to say goodbye.” “I have a dog who recently passed the PP [Pet Partners] evaluation, so we expect to be working together in the fall.”
Support From Friends and Family	“In general, everyone has been very sympathetic about retirement/loss.” “We had children write letters to one dog that died.” “Staff were very affected by the loss as well.”
Memorials, Validation, and Recognition of the Therapy Dog’s Work	“I am proud to remember my dog’s 11 years of service, thinking of her memorable encounters.” “In our group we write an article about our lost partner… It helps.” “Sharing my dog’s life story on social media, posting photos, and receiving kind messages from friends and loved ones.”
Peer Support/Talking with Others Who Had Similar Losses	“Have friends who have been in your situation—helps.” “Everyone has been very sympathetic about retirement/loss.”

**Table 4:** Reasons for Retiring a Therapy Dog.

Retirement Reason (*n* = 83) *	Frequency	Percent
My dog’s old age	46	55.4%
My dog’s medical issues	36	43.4%
My dog didn’t seem to enjoy it anymore	21	25.3%
Behavioral or stress-related issues	14	16.9%
Other reasons	10	12.0%
My health issues	5	6.0%
Lifestyle changes (new job, family responsibilities, etc.)	4	4.8%
I no longer had the opportunity (space, time, etc.)	1	1.2%

* Multiple responses allowed.

**Table 5:** Thematic Analysis of Handlers’ Perceived Supports Following Therapy Dog Retirement.

Theme	Representative Quotes
Having Another Dog/Succession Planning	“I always have 2 working dogs, so I keep working even if one retires.” “Have another dog to take over.” “Another dog to train but I have Jupiter—I just don’t want to overwhelm him.”
Support From Friends and Family	“Conversation with friends.” “My family and fellow AAI volunteers have always embraced, supported and celebrated my fluffs.” “My husband.”
Support From AAI Peers/Professional Community	“Support group of others who have had to retire their therapy dog partnership.” “Support from the AAT community, especially hearing stories through webinars.” “People who understand how sad and hard it is and how much I doubt my decision.”
Confidence in the Decision/Internal Reassurance	“Reassurance from the vet it was time.” “I was confident it was the right decision.” “My dog was not enjoying volunteering… lots of good memories made it easier.”
Recognition, Memorialization, and Validation	“The hospital my dog worked at threw her a retirement party—it was wonderful.” “They continued to include her in recognitions… it was very meaningful that she was not forgotten.” “I had to figure out ways to inform clients… stories, pictures, and an app, My Talking Pet.”
Gradual Transition/Structured Retirement Planning	“Flexibility to make the transition gradually.” “Slowly transitioning a dog partner into retirement instead of abruptly.” “Having a retirement plan in place before we reached that point.”
Staying Involved in AAI Without a Dog	“I stay active in our group without an animal while training a new partner.” “Being able to continue working with my older therapy dog so it wasn’t a complete loss.” “He unofficially visits friends in care homes and children who need comfort.”

## Data Availability

The data presented in this study are available on request from the corresponding author.

## Supplementary Materials

The following supporting information can be downloaded at: https://www.mdpi.com/article/10.3390/ani16020202/s1, Table S1: Characteristics of Animal-Assisted Service (AAS) Volunteers and Their Dog Partners (*n* = 247).

## References

[1] Charry-Sánchez J.D., Pradilla I., Talero-Gutiérrez C. (2018). Animal-assisted therapy in adults: A systematic review. Complement. Ther. Clin. Pract..

[2] Friedman E. (2025). The Animal–Human Bond: Health and Wellness. Handbook on Animal-Assisted Therapy.

[3] Lundqvist M., Carlsson P., Sjödahl R., Theodorsson E., Levin L.-Å. (2017). Patient benefit of dog-assisted interventions in health care: A systematic review. BMC Complement. Altern. Med..

[4] Waite T.C., Hamilton L., O’Brien W. (2018). A meta-analysis of animal-assisted interventions for trauma. Complement. Ther. Clin. Pract..

[5] Binder A.J., Parish-Plass N., Kirby M., Winkle M., Skwerer D.P., Ackerman L., Brosig C., Coombe W., Delisle E., Enders-Slegers M.-J. (2024). Recommendations for uniform terminology in animal-assisted services (AAS). Hum.-Anim. Interact..

[6] Fine A.H., Griffin T.C. (2022). Protecting Animal Welfare in Animal-Assisted Intervention: Our Ethical Obligation. Semin. Speech Lang..

[7] Gee N., Fine A., Kaufman M. (2025). Animals in Educational Settings Research and Practice. Handbook on Animal-Assisted Therapy.

[8] Glenk L.M. (2017). Current Perspectives on Therapy Dog Welfare in Animal-Assisted Interventions. Animals.

[9] Jegatheesan B., Beetz A., Ormerod E., Choi G., Dudzik C., Fine A., Garcia R.M., Johnson R., Winkle M., Yamazaki K. (2014). The IAHAIO Definitions for Animal Assisted Intervention and Guidelines for Wellness of Animals Involved. https://iahaio.org/wp/wp-content/uploads/2017/05/iahaio-white-paper-final-nov-24-2014.pdf.

[10] Vierimaa J., Pyyhtinen O. (2025). Multispecies Interaction in Dog-Assisted Therapy Sessions. Theory Cult. Soc..

[11] Chandler C.K. (2018). Human-animal Relational Theory: A Guide for Animal-assisted Counseling. J. Creat. Ment. Health.

[12] Jegatheesan B. (2018). The IAHAIO Definitions for Animal Assisted Intervention and Guidelines for Wellness of Animals Involved in AAI.

[13] Deci E.L., Ryan R.M. (2015). Self-Determination Theory. International Encyclopedia of the Social & Behavioral Sciences.

[14] Callero P.L. (1985). Role-Identity Salience. Soc. Psychol. Q..

[15] Kirnan J., Ciarrocca A., Malloy M., Hoehne S., Norris G., Nuzzo M. (2024). “My Dog Needs a Job”: Identifying the Motivations of Therapy Animal Volunteers. People Anim. Int. J. Res. Pract..

[16] Bussolari C., Currin-McCulloch J., Packman W., Kogan L., Erdman P. (2024). The Loss of a Service Dog Through Death: Experiences of Partners. Illn. Crisis Loss.

[17] Kogan L.R., Packman W., Bussolari C., Currin-McCulloch J., Erdman P. (2022). Pet Death and Owners’ Memorialization Choices. Illn. Crisis Loss.

[18] Kogan L.R., Packman W., Currin-McCulloch J., Bussolari C., Erdman P. (2023). The Loss of a Service Dog Through Death or Retirement: Experiences and Impact on Partners. Illn. Crisis Loss.

[19] Messam L.L.M., Hart L.A., Kogan L., Blazina C. (2019). Chapter 15—Persons Experiencing Prolonged Grief After the Loss of a Pet. Clinician’s Guide to Treating Companion Animal Issues.

[20] Packman W., Field N.P., Carmack B.J., Ronen R. (2011). Continuing Bonds and Psychosocial Adjustment in Pet Loss. J. Loss Trauma.

[21] Boss P. (2006). Loss, Trauma, and Resilience: Therapeutic Work with Ambiguous Loss.

[22] Boss P. (2007). Ambiguous Loss Theory: Challenges for Scholars and Practitioners. Fam. Relat..

[23] Boss P. (1999). Ambiguous Loss: Living with Frozen Grief. Harv. Ment. Health Lett..

[24] Boss P., Yeats J.R. (2014). Ambiguous loss: A complicated type of grief when loved ones disappear. Bereave. Care.

[25] Currin-McCulloch J., Bussolari C., Packman W., Kogan L., Erdman P. (2022). The Loss of a Service Dog Through Retirement: Experiences and Impact on Human Partners. Hum.-Anim. Interact. Bull..

[26] Lepore S.J., Revenson T.A. (2007). Social Constraints on Disclosure and Adjustment to Cancer. Soc. Personal. Psychol. Compass.

[27] Beinke K.L., O’Callaghan F.V., Morrissey S. (2015). The impact of social constraints and sense of coherence on the psychological adjustment of adolescents and young adults with CF. Cogent Psychol..

[28] Cordova M.J., Cunningham L.L.C., Carlson C.R., Andrykowski M.A. (2001). Social constraints, cognitive processing, and adjustment to breast cancer. J. Consult. Clin. Psychol..

[29] Lepore S.J. (2001). A social–cognitive processing model of emotional adjustment to cancer. Psychosocial Interventions for Cancer.

[30] Manne S., Ostroff J., Rini C., Fox K., Goldstein L., Grana G. (2004). The Interpersonal Process Model of Intimacy: The Role of Self-Disclosure, Partner Disclosure, and Partner Responsiveness in Interactions Between Breast Cancer Patients and Their Partners. J. Fam. Psychol..

[31] Rivera J.N., Burris J.L. (2020). A Systematic Literature Review and Head-to-Head Comparison of Social Support and Social Constraint in Relation to the Psychological Functioning of Cancer Survivors. Ann. Behav. Med..

[32] Chur-Hansen A. (2010). Grief and bereavement issues and the loss of a companion animal. Clin. Psychol..

[33] Morley C., Fook J. (2005). The importance of pet loss and some implications for services. Mortality.

[34] Park R.M., Royal K.D., Gruen M.E. (2023). A Literature Review: Pet Bereavement and Coping Mechanisms. J. Appl. Anim. Welf. Sci..

[35] Volsche S. (2019). Pet Parents and the Loss of Attachment. Pet Loss, Grief, and Therapeutic Interventions.

[36] Doka K.J. (1999). Disenfranchised grief. Bereave. Care.

[37] Grube J.A., Piliavin J.A. (2000). Role Identity, Organizational Experiences, and Volunteer Performance. Pers. Soc. Psychol. Bull..

[38] Thoits P.A. (2012). Role-Identity Salience, Purpose and Meaning in Life, and Well-Being among Volunteers. Soc. Psychol. Q..

[39] Gibson M., Chalmers D., Ru S. (2022). “My Lifeline is Gone”: An Exploration of the Experiences of Veterans Following the Loss of their Psychiatric Service Dog(s). Hum.-Anim. Interact. Bull..

[40] Knoll T.E., Bould E., Callaway L., Iannos M. (2025). Planning for the retirement or death of an assistance dog: Perspectives of staff from assistance animal organisations. Disabil. Rehabil. Assist. Technol..

[41] Ng Z., Fine A. (2019). Paving the Path Toward Retirement for Assistance Animals: Transitioning Lives. Front. Vet. Sci..

[42] Worth A.J., Sandford M., Gibson B., Stratton R., Erceg V., Bridges J., Jones B. (2013). Causes of loss or retirement from active duty for New Zealand police German shepherd dogs. Anim. Welf..

[43] Yamamoto M., Hart L.A. (2019). Separation from Assistance Dogs: The Complicated Psychological Burden During Loss of the Relationship. Pet Loss, Grief, and Therapeutic Interventions.

[44] Mc Veigh M.J. (2023). “Giving voice to the voiceless”: An exploration of the grieving ritual for a therapy dog. Death Stud..

[45] Barberi D., Gibbs J.C., Schally J.L. (2019). K9s killed in the line of duty. Contemp. Justice Rev..

[46] Wise J., Graham A., Dodge C., Somers L.J. (2025). Police K-9 line-of-duty deaths and heatstroke 2000–2023. Police Pract. Res..

[47] O’Connor M.-F., Wellisch D.K., Stanton A.L., Olmstead R., Irwin M.R. (2012). Diurnal cortisol in Complicated and Non-Complicated Grief: Slope Differences across the Day. Psychoneuroendocrinology.

[48] Seiler A., von Känel R., Slavich G.M. (2020). The Psychobiology of Bereavement and Health: A Conceptual Review from the Perspective of Social Signal Transduction Theory of Depression. Front. Psychiatry.

[49] Alotiby A. (2024). Immunology of Stress: A Review Article. J. Clin. Med..

[50] James K.A., Stromin J.I., Steenkamp N., Combrinck M.I. (2023). Understanding the relationships between physiological and psychosocial stress, cortisol and cognition. Front. Endocrinol..

[51] Yaribeygi H., Panahi Y., Sahraei H., Johnston T.P., Sahebkar A. (2017). The impact of stress on body function: A review. EXCLI J..

[52] Lepore S.J., Silver R.C., Wortman C.B., Wayment H.A. (1996). Social constraints, intrusive thoughts, and depressive symptoms among bereaved mothers. J. Personal. Soc. Psychol..

[53] Braun V., Clarke V. (2006). Using thematic analysis in psychology. Qual. Res. Psychol..

[54] Erdman P., Ruby K. (2019). Grieving pet loss. Pet Loss, Grief, and Therapeutic Interventions: Practitioners Navigating the Human-Animal Bond.

[55] McAdams D.P., McLean K.C. (2013). Narrative Identity. Curr. Dir. Psychol. Sci..

[56] McDonald S.E., Kogan L.R., Nageotte N.L., Currin-McCulloch J., Dickler-Mann R. (2024). Zoo professionals and volunteers in the U.S: Experiences and prevalence of burnout, mental health, and animal loss. Front. Psychiatry.

[57] Neimeyer R.A. (2016). Meaning Reconstruction in the Wake of Loss: Evolution of a Research Program. Behav. Change.

[58] Romanoff B.D., Terenzio M. (1998). Rituals and the grieving process. Death Stud..

[59] Disanayake M., Howell T., Oliva J. (2023). From Puppy Love to Pet Peeve: What Causes Second/Successor Dog Syndrome in Assistance-Dog Handlers and Companion-Dog Owners?. Anthrozoös.

[60] Kogan L.R., Currin-McCulloch J., Brown E., Hellyer P. (2024). Dog owners’ perceptions and veterinary-related decisions pertaining to changes in their dog’s behavior that could indicate pain. J. Am. Vet. Med. Assoc..

[61] Miller S.L., Serpell J.A., Dalton K.R., Waite K.B., Morris D.O., Redding L.E., Dreschel N.A., Davis M.F. (2022). The Importance of Evaluating Positive Welfare Characteristics and Temperament in Working Therapy Dogs. Front. Vet. Sci..

[62] Ng Z.Y., Pierce B.J., Otto C.M., Buechner-Maxwell V.A., Siracusa C., Werre S.R. (2014). The effect of dog–human interaction on cortisol and behavior in registered animal-assisted activity dogs. Appl. Anim. Behav. Sci..

[63] Burrell A., Selman L.E. (2022). How do Funeral Practices Impact Bereaved Relatives’ Mental Health, Grief and Bereavement? A Mixed Methods Review with Implications for COVID-19. Omega.

[64] Sas C., Coman A. (2016). Designing personal grief rituals: An analysis of symbolic objects and actions. Death Stud..

[65] Valentine C. (2019). Meaning-making in bereavement and grief. Bereave. Care.

[66] Field N.P., Orsini L., Gavish R., Packman W. (2009). Role of attachment in response to pet loss. Death Stud..

[67] Neimeyer R., Baldwin S.A., Gillies J. (2006). Continuing Bonds and Reconstructing Meaning: Mitigating Complications in Bereavement. Death Stud..

