# Supplementary material for: Navigating Loss in Animal-Assisted Services: Volunteer Experiences and Implications for Programs Following Therapy Dog Death or Retirement
Source: Animals (Basel). 2026 Jan 9;16(2):202. doi: 10.3390/ani16020202 (PMC12837273; doi:10.3390/ani16020202)
Supplement: Supplementary file 1 [file animals-16-00202-s001.zip › animals-4057391-supplementary.pdf]

Supplemental Materials

Table S1. Characteristics of Animal-Assisted Service (AAS) Volunteers and Their Dog Partners (N = 247).

**Variable**	**Category**	**Frequency**	**Percent (%)**
Currently providing AAS	Yes	202	81.8
	No, but have in the past	45	18.2
Number of AAS dogs personally trained	0	4	1.6
	1	112	45.3
	2	53	21.5
	3	18	7.3
	4	17	6.9
	5 or more	43	17.4
Number of currently engaged AAS dogs	0 dogs	37	15.0
	1 dog	163	66.0
	2 dogs	34	13.8
	3 dogs	8	3.2
	5 or more	4	1.6
Acquired dog with AAS intent	Yes	103	41.7
	No	96	38.9
	Unsure	11	4.5
Dog certified/registered as therapy dog	Yes	191	77.3
	No, but in process	8	3.2
	No	11	4.5
